# Applying a PID-SMC synthetic control algorithm to the active suspension system to ensure road holding and ride comfort

**DOI:** 10.1371/journal.pone.0283905

**Published:** 2023-10-19

**Authors:** Tuan Anh Nguyen

**Affiliations:** Thuyloi University, Hanoi, Vietnam; University of Hull, UNITED KINGDOM

## Abstract

Vehicle vibration has an essential effect on vehicle stability and smoothness. This article introduces a new solution to direct an active suspension system called the PID-SMC hybrid algorithm. In this work, a dynamic model is considered with external disturbances and parametric uncertainties. Besides, the design process of the hybrid controller is also clearly shown. Different from previous studies, this controller is built upon the synthesis of two component signals which are generated from two separate controllers. The error signals of the two-component controllers are derived from the results of the body displacement and acceleration measured by the sensors. A simulation process is done by the MATLAB software to evaluate the system’s quality. Two cases are used for the simulation, including four scenarios examined for each case. Based on the results obtained from the simulation and calculation technique, the acceleration and displacement values of a vehicle body were greatly decreased once the PID-SMC method was used, compared with the rest of situations. In the first case, the maximum value of the acceleration is only 0.54 (m/s^2^), while the average value and the RMS value are 0.06 (m/s^2^) and 0.07 (m/s^2^), respectively. In the second case, the maximum value of vehicle body displacement is only 9.54 (mm), only 8.75%, compared to cars with only mechanical suspensions. Besides, the change in the dynamic load at the wheel is also relatively small. Therefore, the road holding and ride comfort of the automobile has been improved. In the near future, this algorithm will be combined with intelligent control algorithms to apply to many different types of random stimuli from the road surface.

## 1. Introduction

Vibrations of an automobile are caused by many reasons, in which bad road surface conditions are the important cause of this problem. Under each specific condition, the frequency and amplitude of the excitation signal are different. So, the vibration of the automobile in different cases is different. Vehicle vibration is a serious problem and can negatively affect the comfort and smoothness of the automobile when moving. If these vibrations occur continuously for a long time, the health of passengers may be negatively affected. To minimize the vibrations of the vehicle when moving, the suspension system must be equipped in all cars today. A typical suspension system found on most vehicles is the passive suspension system (mechanical). For a passive suspension, the spring’s stiffness and damping are unchanged. Therefore, the smoothness of an automobile when utilizing a conventional mechanical suspension system is not high. The first solution to overcome this situation is using a suspension system that can flexibly change the stiffness. In [1], Zhao et al. introduced an air suspension system with multiple automobile uncertainties. The supply of compressed air is controlled by solenoid valves, which are fitted to the air balloons of the suspension system. A suspension system’s stiffness depends on the compressed air pressure inside the balloons, and it will affect the ride comfort during vibration [2]. Another solution is to use electromagnetic absorbers, which operate on the principle of electromagnetic force. The suspension system using electromagnetic damping is also known as semi-active suspension [3]. According to Sande et al., the unsprung mass acceleration calculated according to the RMS (Root Mean Square) has decreased by 11% [4]. Although both solutions help reduce the vibration of the car; however, their effect is not so good because they can only change the stiffness of one part of the suspension system. If it is possible to equip an additional component that actively generates a force on the unsprung and sprung mass, ride comfort can be enhanced. Therefore, active hydraulic suspension incorporating a hydraulic actuator is used in several vehicles today [5]. The hydraulic actuator of an active hydraulic suspension system will be directed by the controller, which has been previously designed [6]. The operation of the actuator will entirely depend on the system’s control method.

Several studies on suspension system control have been published in the last few years. In [7], Abdullah et al. introduced using the MOPID (Multi-Object Proportional-Integral-Derivative) algorithm for a suspension system. According to classical control theory, the PID method only should apply to the SISO (Single-Input Single-Output) system, so only one object can be controlled using this algorithm. However, if more objects need to be controlled, the PID algorithm can be extended to become MOPID. It can be understood simply that this is a combination of three single PID controllers. The parameters of this controller can be tuned by the CSD (Control System Designer) app, which Shafiei introduced in [8]. Another solution to tune the parameters of the PID controller is using the Fuzzy algorithm [9] or GA (Genetic Algorithm) [10]. In [11], Dong et al. improved the traditional PID algorithm to become an advanced FOPID (Fractional Order Proportional-Integral-Derivative) algorithm. According to Swethamarai and Lakshmi, the FOPID algorithm has many parameters which need to be adjusted more than the PID algorithm [12]. If these parameters are selected appropriately, the controller’s quality will improve. For systems with many objects that need to be controlled, the LQR (Linear-Quadratic Regulator) algorithm will be a suitable solution [13]. According to Nguyen et al., the control signal of the LQR algorithm can be calculated based on a matrix, which is calculated according to the Riccati algebraic equation. This algorithm’s ultimate aim is to optimize the controller’s cost function [14]. The coefficients of the state matrix can be selected by the in-loop optimization method [14] or the bat optimization algorithm [15] to increase the system’s quality.

The above methods are only suitable for linear systems. For nonlinear oscillating systems, applying other, more complex algorithms is necessary. In [16], Qin et al. propose using a Robust control solution for a suspension system. This algorithm is established based on the theory of H_2_ and H_∞_ with oscillating state matrices [17]. Today, the Robust control algorithm can be applied to the suspension system model of passenger cars, heavy trucks, and electric vehicles [18, 19]. For systems oscillating continuously under different conditions, an Adaptive control algorithm is a suitable choice [20, 21]. A typical method commonly used to control nonlinear systems is the SMC (Sliding Mode Control) algorithm [22]. This algorithm can be applied to highly efficient linear and nonlinear systems [23]. According to [24], a SMC control method must take the high-order derivative of the output signal so that the order of the derivative must be equal to the number of state variables. For a quarter dynamic model with an actuator included, the number of state variables is 5. This number can be increased to 10 if a half-dynamic model with two separate hydraulic actuators is used. However, the quality of individual controls still cannot meet the requirements set for the vibration and stability of the car. Therefore, it is necessary to combine component controllers to create an adaptive controller suitable for the oscillating conditions of the suspension system.

Complex control algorithms can be combined to enhance the system’s quality. In [25], Zhang and Jing used an integrated controller, a combination of the Fuzzy and SMC algorithms. Hsiao and Wang again showed this combination in their study [26]. Another combination, such as PD-SMC, was performed by Zhang et al. [27] and gave high efficiency. In addition, Nguyen and Nguyen presented combining SMC and PID controllers for multivariate models [28]. Compared with single control algorithms, integrated algorithms usually yield higher quality. In addition, some intelligent control algorithms have been utilized to direct an active suspension system in an automobile [29–31]. The above studies almost use the excitation from the pavement as the input signal for a simulation problem. The output signal can include the displacement and acceleration of an automobile. These values can be expressed in the frequency domain [32] or the time domain [33]. In addition to the modern SMC algorithm, several other effective ways to control nonlinear systems, such as robust back-stepping control [34], adaptive FIT-SMC control [35], etc. Active suspension systems can make the ride more comfortable by generating impact forces. However, the wheel may stop interacting with the road surface if these forces are too strong. According to Ahmad et al., a balance between road holding and the ride comfort is necessary [36]. Besides, several other control methods for nonlinear systems that consider the effects of noise, uncertainties, delays, and constraints are also applied to mechatronic systems [37, 38]. These achievements can be applied to the active suspension model in the future.

As mentioned above, replacing single controls with integrated ones is essential to help ensure suspension stability. In this article, an advanced control algorithm will be used in order to direct an active suspension system. This integration algorithm is a combination of PID algorithm and SMC algorithm. The final control signal of the controller will be synthesized from the component control signals. The input of the PID controller and the SMC controller are two separate signals that can be obtained from the sensor. The coefficients are selected based on criteria related to both ride comfort and road holding, which are essential, according to Ahmad et al. [36]. This is considered a new point of the article compared to previous studies, which usually only focus on ride comfort. The introduction and review of the research topic are given in the Introduction section. The design process of the dynamic model and control algorithm can be further clarified in the Model and Algorithm section. The calculation and simulation will be done with specific conditions in the next section. Finally, some essential points will be pointed out in the Conclusions section.

## 2. Model and algorithm

**Fig 1:**
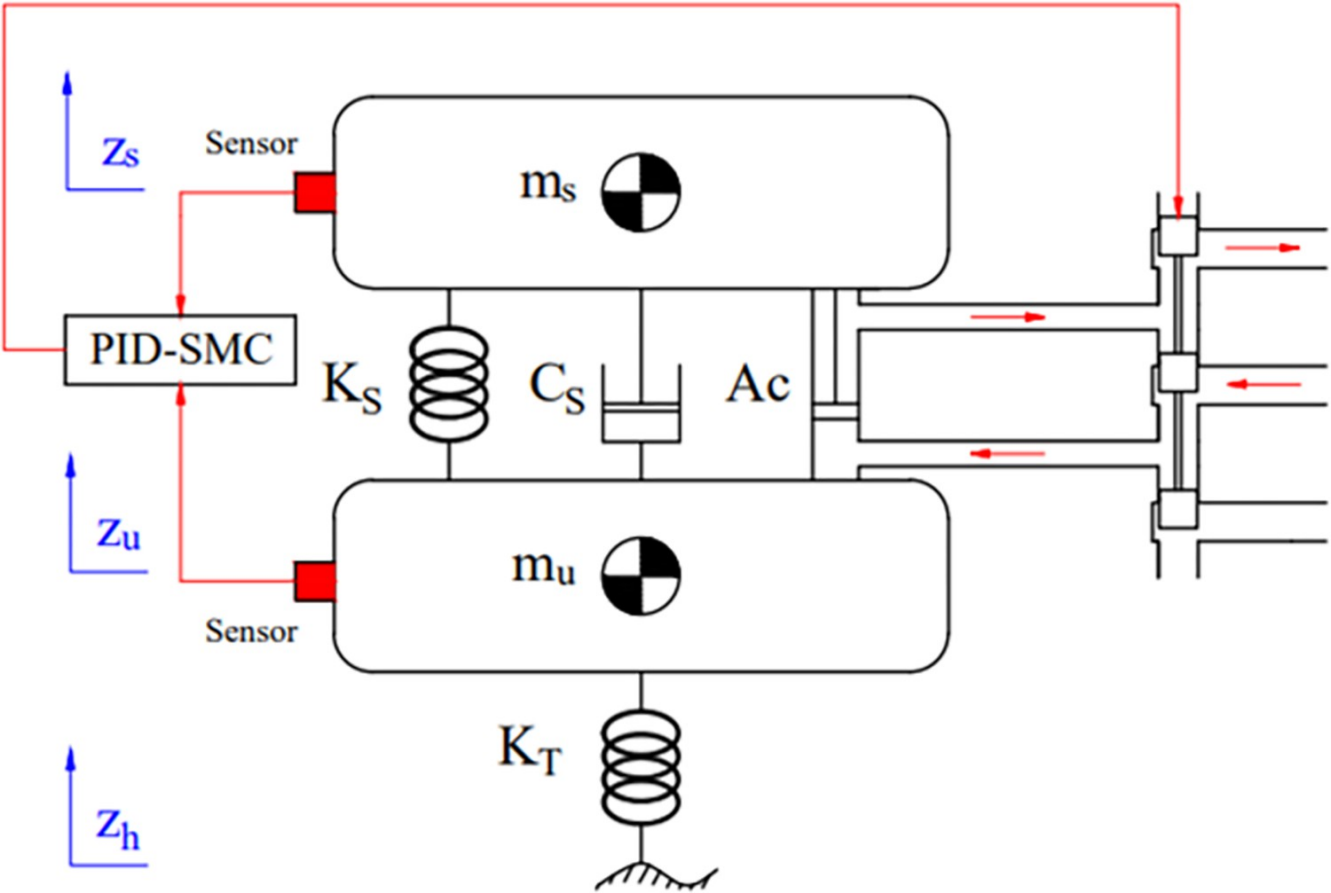
Model of the quarter-dynamics.

Vibrations in a vehicle may be simulated using a variety of dynamics models. A model with quarter dynamics is frequently employed when dealing with control issues. The hydraulic actuator is an integral part of this type (Fig 1). The excitation from the road surface is the cause of oscillation, which is transmitted directly to the wheels. These vibrations are then transmitted to the vehicle body through the suspension system. This system has the role of controlling and quenching oscillations. For active suspension, the hydraulic actuator generates an impact force transmitted to the sprung and unsprung masses for vibration control.

Separate the mass into its sprung and unsprung parts. This gives it two displacements, *z*_*s*_ (for *m*_*s*_) and *z*_*u*_ (for *m*_*u*_), corresponding to two degrees of freedom. Using the D’alembert principle, we obtain two equations describing the motion of 2 masses.

For the sprung mass (vehicle body):

$$m_{s}{\ddot{z}}_{s}=F_{KS}+F_{CS}+F_{Ac}$$ (1)

For the unsprung mass:

$$m_{u}{\ddot{z}}_{u}=F_{KT}-F_{KS}-F_{CS}-F_{Ac}$$ (2)

Where:

Spring force:

$$F_{KS}=K_{S}(z_{u}-z_{s})$$ (3)

Damper force:

$$F_{CS}=C_{S}({\dot{z}}_{u}-{\dot{z}}_{s})$$ (4)

Tire force:

$$F_{KT}=K_{T}(z_{h}-z_{u})$$ (5)

The Laplace transform of the above equations, the system’s oscillation description state-space matrix (PLANT), is rewritten in the following form:

$$\left[\begin{array}{c} Z_{s}(s)\\ Z_{u}(s) \end{array}]=\frac{1}{A}[\begin{array}{cc} B_{11} & B_{12}\\ B_{21} & B_{22} \end{array}][\begin{array}{c} F_{Ac}(s)\\ Z_{h}(s) \end{array}\right]$$ (6)

Where:

$$A=(m_{s}s^{2}+C_{S}s+K_{S})(m_{u}s^{2}+C_{S}s+K_{S}+K_{T})-(C_{S}s+K_{S})(C_{S}s+K_{S})$$ (7)

$$B_{11}=M_{u}s^{2}+K_{T}$$ (8)

$$B_{12}=C_{S}K_{T}s+K_{S}K_{T}$$ (9)

$$B_{21}=-m_{s}s^{2}$$ (10)

$$B_{22}=m_{s}K_{T}s^{2}+C_{S}K_{T}s+K_{S}K_{T}$$ (11)

The actuator’s output impact force may be approximately calculated using the following the linear Eq (12) [24].


$${\dot{F}}_{Ac}=\gamma_{1}u(t)-\gamma_{2}F_{Ac}-\gamma_{3}({\dot{z}}_{u}-{\dot{z}}_{s})$$ (12)

For the active suspension model, the stimuli from the road surface that cause vehicle vibration are considered uncertainties. These stimuli are taken from outside that change over time, such as sinusoidal, step, random, etc. Besides, other uncertainties can also be mentioned, such as wind, powertrain fluctuations, etc. (these can be ignored if their effect is insignificant).

The dynamic model is a time-invariant linear model, but the input stimuli have a nonlinear form that varies with time. It is not reasonable to use only the traditional PID method. Therefore, the classical PID method should be combined with another solution suitable for controlling nonlinear systems. As a result of the fact that the object is nonlinear, it has been suggested that the SMC method be used in this model. The SMC control strategy can be found in [39]. The SMC algorithm can cause the "chattering" phenomenon, negatively affecting the system’s quality. This problem can be solved using the state-dependent Kalman filter mentioned by Ahmad et al. [40].

Let’s say that the error between the setpoint signal *y*_*s1*_*(t)* and the output signal *y(t)* is denoted by the variable *e*_*1*_*(t)*:

$$e_{1}(t)=y_{s1}(t)-y(t)$$ (13)

**Fig 2:**
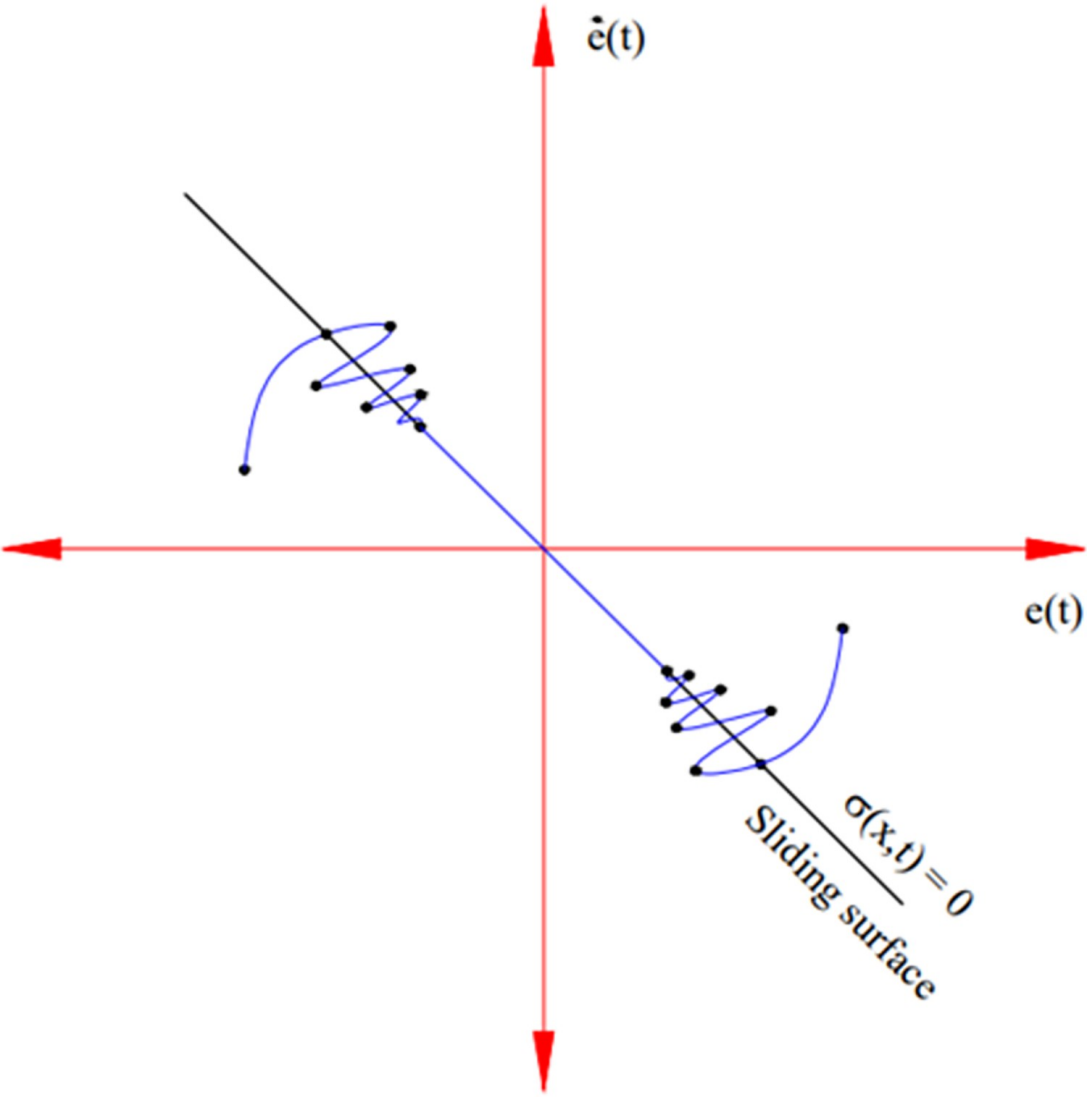
Sliding surface.

The error signal *e*_*1*_*(t)* needs to become zero before the system can be considered stable. This value will oscillate around the sliding surface and move towards a stable position (Fig 2). In order to make the problem easier to understand, a linear sliding surface is frequently utilized.


$$s(e)=a_{0}e+a_{1}\frac{de}{dt}+a_{2}\frac{d^{2}e}{dt^{2}}\ldots +a_{n-2}\frac{d^{n-2}e}{dt^{n-2}}+\frac{d^{n-1}e}{dt^{n-1}}$$ (14)

It is necessary for the coefficients *a*_*i*_ (from *a*_*0*_ to *a*_*n-2*_) of the sliding surface *s(e)* (14) are coefficients of the polynomial *P(p)* in such a way that the resulting this polynomial is a Hurwitz polynomial (15).


$$P(p)=a_{0}+a_{1}p+a_{2}p^{2}+\ldots +a_{n-2}p^{n-2}+p^{n-1}$$ (15)

The Formula (16) demonstrates the requirements for the systematic error to become zero. The sliding condition of the control problem describes this problem.


$$\frac{ds}{dt}sgn(s)<0$$ (16)

Take the following model for an n-order nonlinear object:

$$\left\{ \begin{array}{l} \frac{dx_{1}}{dt}=x_{2}\\ \frac{dx_{2}}{dt}=x_{3}\\ \ldots \\ \frac{dx_{n-1}}{dt}=x_{n}\\ \frac{dx_{n}}{dt}=f(x_{1},x_{2},\ldots ,x_{n})+u_{1}(t) \end{array}\right.$$ (17)

Suppose the model’s output is a form of state variable:

$$y_{1}(t)=x_{1}$$ (18)

The sliding surface *s(e)* is rewritten as:

$$s(e)=\sum_{i=0}^{n-1}a_{i}\frac{d^{i}e}{dt^{i}}=\sum_{i=0}^{n-1}a_{i}\frac{d^{i}y_{s1}}{dt^{i}}-\sum_{i=0}^{n-1}a_{i}\frac{d^{i}y}{dt^{i}}$$ (19)

Let:

$$\tilde{y_{s1}}=\sum_{i=0}^{n-1}a_{i}\frac{d^{i}y_{s1}}{dt^{i}}$$ (20)

The sliding surface (19) becomes:

$$s(e)=\tilde{y_{s1}}-\sum_{i=0}^{n-1}a_{i}x_{i+1}$$ (21)

Substitute (21) into (16), get:

$$(\frac{d\tilde{y_{s1}}}{dt}-\sum_{i=0}^{n-1}a_{i}\frac{dx_{i+1}}{dt})sgn(s)<0$$ (22)

If the setpoint signal *y*_*s1*_ is unchanged, ie:

$$\frac{d\tilde{y_{s1}}}{dt}=0$$ (23)

The control signal *u*_*1*_*(t)* has the form:

$$u_{1}(t)=-(\sum_{i=0}^{n-2}a_{i}x_{i+2}+f(x_{1},x_{2},\ldots ,x_{n}))+sgn(s)$$ (24)

**Fig 3:**
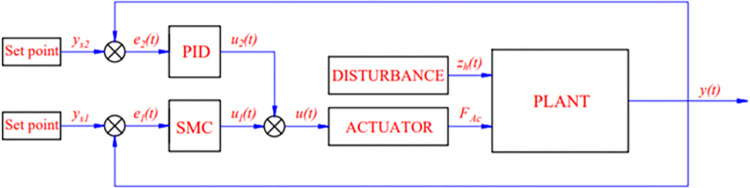
System schematic.

As is evident by looking at Fig 3, the SMC controller is combined with the PID controller to boost the system’s overall performance. The PID controller has several advantages, such as simplicity and high reliability, and is widely applied in practice. Besides, the SMC controller brings stability and robustness to nonlinear systems. When these two controllers are combined, we can get the above benefits. According to Fig 3, the input to the PID controller is the error *e*_*2*_*(t)*, obtained from the set signal *y*_*s2*_ and the output signal *y*_*2*_*(t)*. The input to the SMC controller is the error *e*_*1*_*(t)*, which is obtained from the set signal *y*_*s1*_ and the output signal *y*_*1*_*(t)*. The integrated PID-SMC controller controls *y*_*1*_*(t)* and *y*_*2*_*(t)* output signals more stable. Therefore, this is considered a novel point of the article because it helps to control both outputs to meet the criteria related to road holding and ride comfort.

Let: *e*_*2*_*(t)* be the error signal of the PID controller.


$$e_{2}(t)=y_{s2}(t)-y_{2}(t)$$ (25)

The control signal *u*_*2*_*(t)* is calculated by (26):

$$u_{2}(t)=K_{P}e_{2}(t)+K_{I}\int_{0}^{t}e_{2}(\tau )d\tau +K_{D}\frac{de_{2}(t)}{dt}$$ (26)

Two independent control signals combine to form the integrated controller’s shared control signal.


$$u(t)=u_{1}(t)+u_{2}(t)$$ (27)

After the controller design process is completed, the simulation and calculation process should be carried out. This content will be continued in the following section.

## 3. Simulation

The calculation and simulation process are done to determine the system’s quality. The input of the simulation problem is the excitation signal from the road surface; the output includes the values of sprung mass vertical displacement, sprung mass vertical acceleration, the vertical force at the wheel, etc. Two cases are performed in this work, corresponding to two types of pavement excitation signals. The data used for the simulation process are shown in Table 1.

**Table 1:** Specification parameters.

Notation	Description	Unit	Value
m_s_	Sprung mass	kg	430
m_u_	Unsprung mass	kg	45
K_S_	Spring coefficient	Nm^-1^	42500
C_S_	Damping coefficient	Nsm^-1^	3250
K_T_	Tire coefficient	Nm^-1^	170000

### 3.1. The first case

**Fig 4:**
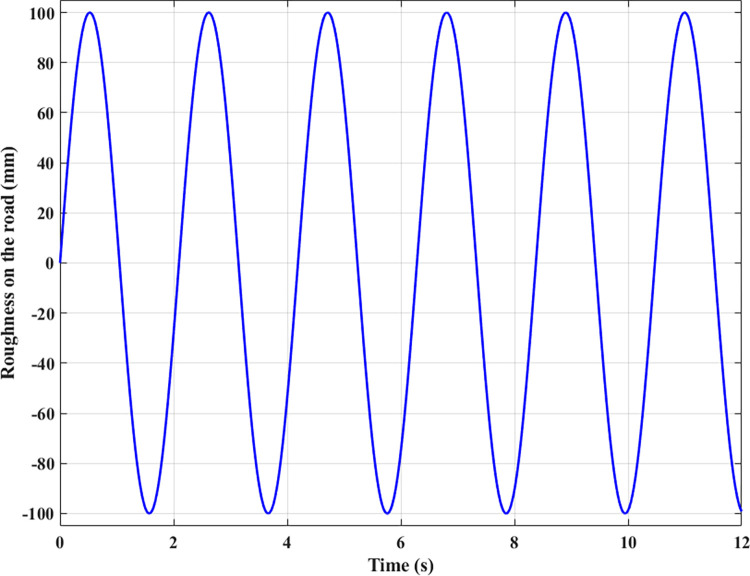
Road surface roughness (the first case).

In the first case, an excitation signal with a sine shape is used. This signal is time-varying with low frequency. The amplitude of the excitation can reach *r(t)* = 100 (mm) (Fig 4).

**Fig 5:**
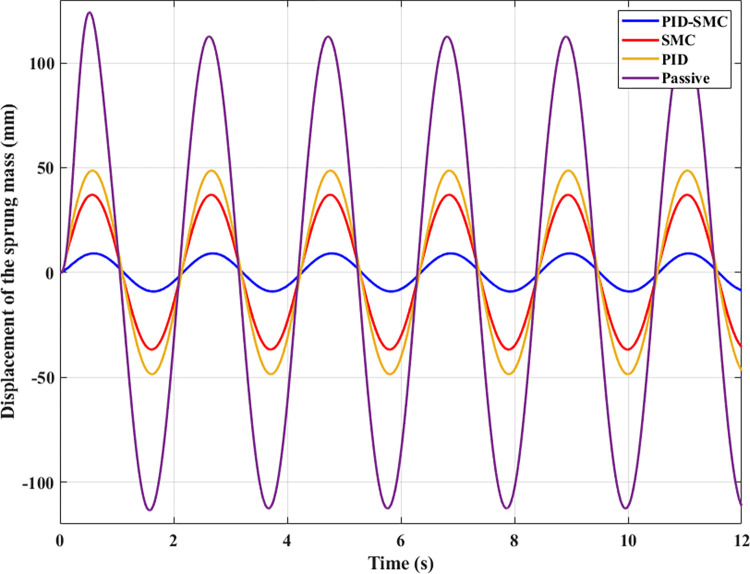
Sprung mass displacement (the first case).

Fig 5 illustrates the change in vehicle body displacement during the investigated period. This change obeys the law of variation of the excitation signal from the road surface. The peak value of vehicle displacement reached 124.13 (mm), 48.65 (mm), 37.08 (mm), and 9.13 (mm), corresponding to four investigated situations, including Passive, PID, SMC, and PID-SMC. The mean value and the root mean square value should also be considered for continuously varying periodic vibrations. The mean value of the vibration can be positive or negative; however, the root mean square value is only positive. According to this result, the mean value of vertical displacement is 71.04 (mm), 30.68 (mm), 23.33 (mm), and 5.73 (mm). Meanwhile, the RMS value is slightly different from the average value, reaching 79.39 (mm), 34.16 (mm), 25.97 (mm), and 6.38 (mm). It is clear that the automobile’s vibration is most remarkable if the automobile has only conventional passive suspension. Conversely, this value can be reduced if the active suspension is utilized to replace the mechanical suspension system. The quality of an active suspension system depends on the control method utilized to direct it. The efficiency of the PID and SMC algorithms is similar (only the first case), while the efficiency of PID-SMC hybrid solution is higher. If the parameters are selected appropriately, the efficiency of the SMC algorithm will be much higher than that of the traditional PID algorithm [41]. However, it still cannot overcome the PID-SMC combination algorithm, demonstrated through simulation results.

**Fig 6:**
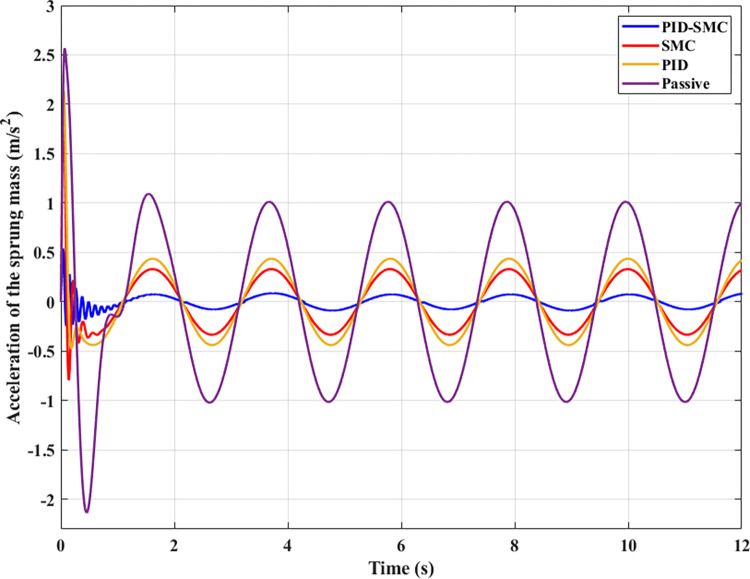
Sprung mass acceleration (the first case).

The sprung mass acceleration is utilized to evaluate the vehicle’s comfort. This result must also be considered with the mean, maximum, and RMS values. The change in vertical acceleration throughout the investigated period is illustrated in Fig 6. Looking at this graph, it can be seen that the value of the vehicle acceleration reaches its maximum at the first phase of the vibration. This happens because the vehicle body suddenly oscillates, so the sprung mass acceleration will peak with an amplitude of up to 2.57 (m/s^2^), 2.13 (m/s^2^), 2.05 (m/s^2^), and 0.54 (m/s^2^) in the same order as above. The difference between the first and last situation can be approximately 4.8 times, considered a large disparity. The acceleration of the vehicle body varies cyclically in a sinusoidal period since the second phase of the vibration. If the automobile does not have the active suspension, a peak value of acceleration is able to be up to about 1.00 (m/s^2^). In contrast, this value in the case of vehicle use, an active suspension vehicle directed by the hybrid solution, is less than 0.1 (m/s^2^). For all four investigated scenarios, the average value of acceleration reached 0.68 (m/s^2^), 0.29 (m/s^2^), 0.22 (m/s^2^), and 0.06 (m/s^2^), respectively. Besides, the root mean square value is also shown by the simulation process, respectively 0.79 (m/s^2^), 0.34 (m/s^2^), 0.27 (m/s^2^), and 0.07 (m/s^2^). The most significant difference belongs to two situations: Passive and PID-SMC; however, the difference between two situations PID and SMC are smaller. With the above results, the ride comfort of the automobile can be improved if the automobile has an active suspension system directed by the PID-SMC hybrid solution.

**Fig 7:**
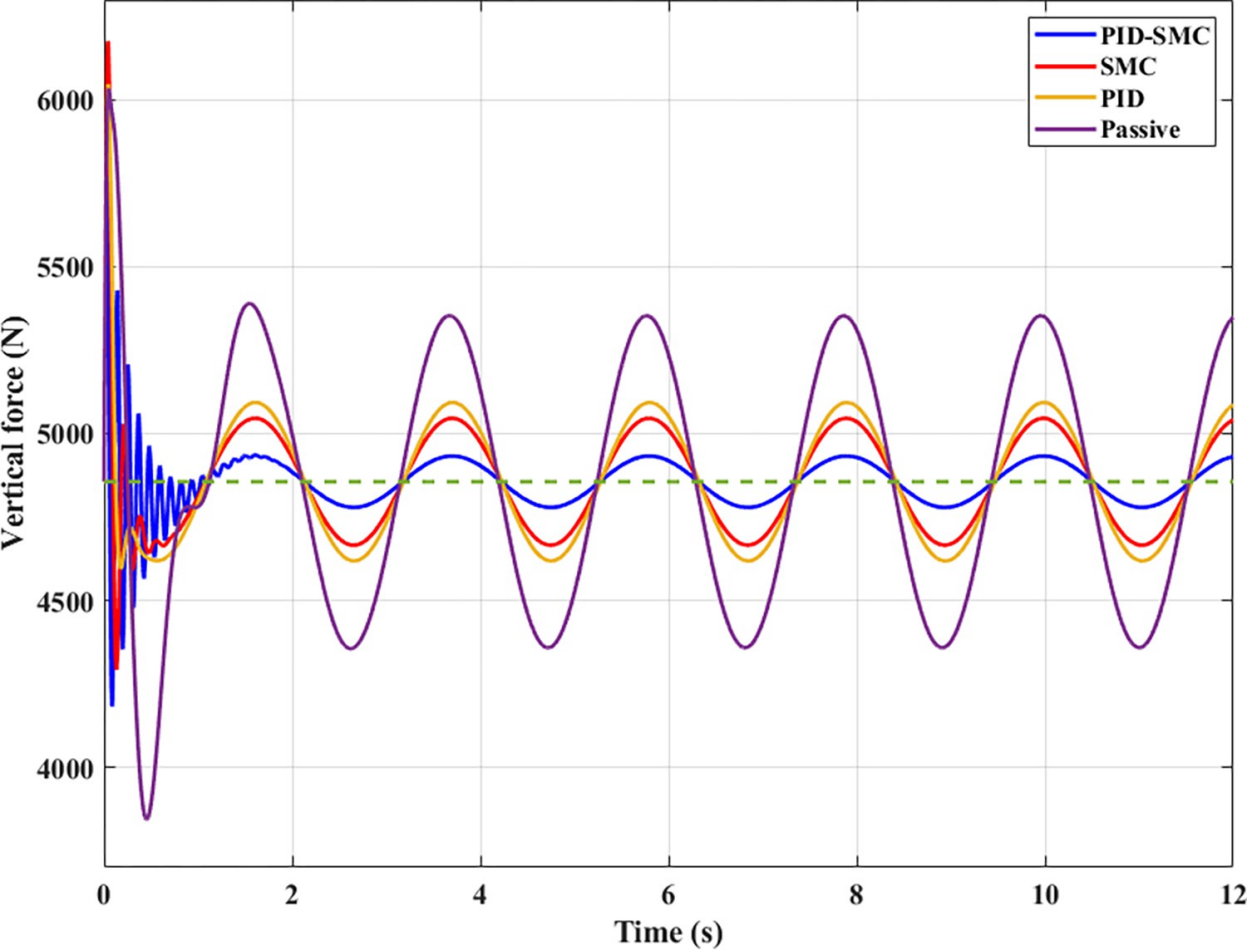
The wheel dynamic load (the first case).

The sprung mass acceleration and displacement values can only evaluate the vehicle’s comfort. So, it is necessary to calculate the change of the dynamic force at the wheel to assess the vehicle’s ability to hold the road when it fluctuates. The change of the dynamic load in the first case is depicted in Fig 7 with four specific situations. According to this result, the change in the dynamic load has the same form as the change in vehicle body acceleration. The most change belongs to the Passive situation; the slightest change is actual for the PID-SMC situation. Because the excitation signal has only a low frequency, the variation of the dynamic load of all situations is not too much. So, the wheel still has good road holding.

This is a typical case often mentioned in problems related to the control of the suspension system. The controller’s stability can be more comprehensively evaluated with timed periodic excitations. So, this case can be considered for future experiments. Excitation from the pavement is described via a dedicated set of rollers, while the sensors measure output values directly. Some of the parametric disturbances should be taken into account when conducting experiments.

### 3.2. The second case

**Fig 8:**
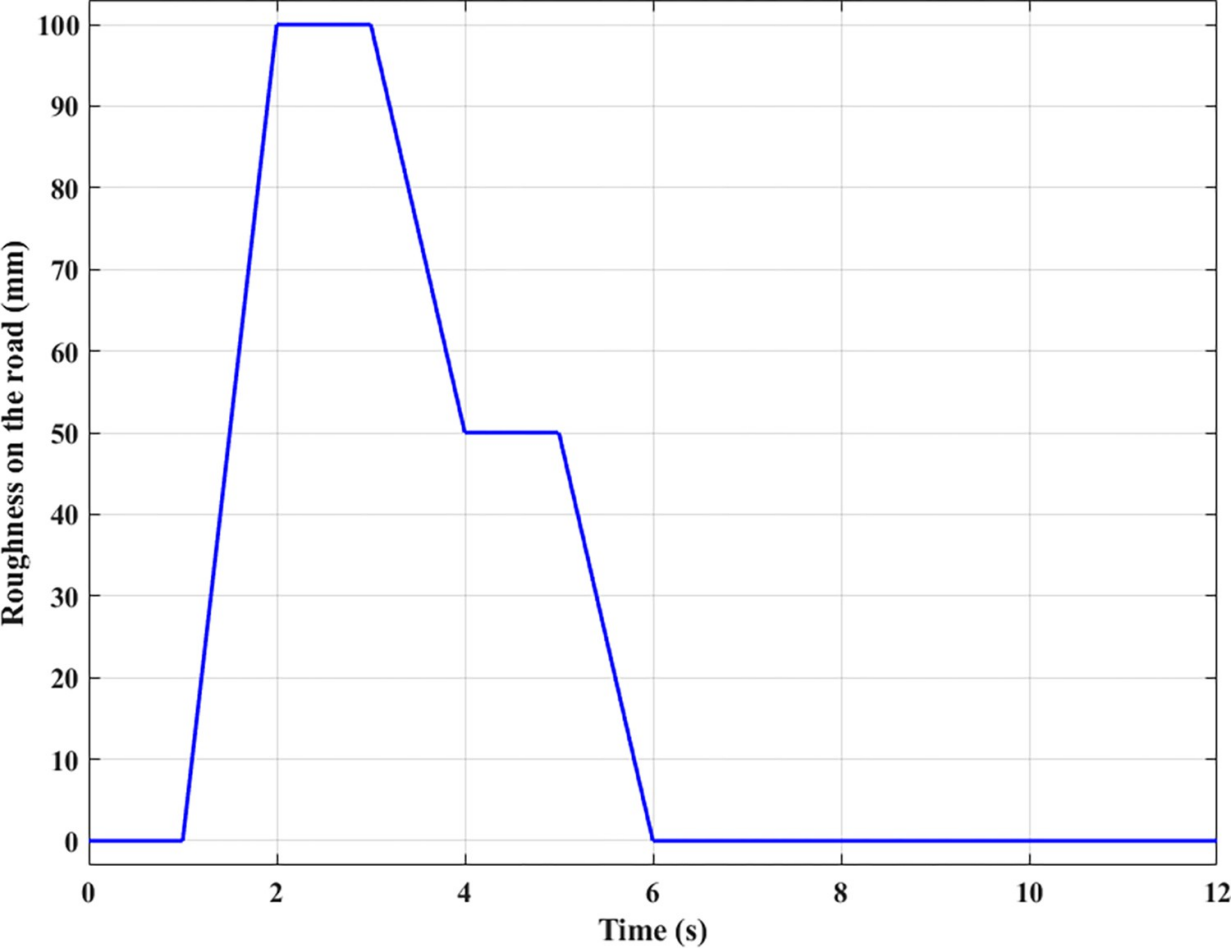
Road surface roughness (the second case).

In the second case, the pavement excitation forms a multistep signal (Fig 8). The values for vehicle body displacement, vehicle body acceleration, and the dynamic load at the wheel are still considered.

**Fig 9:**
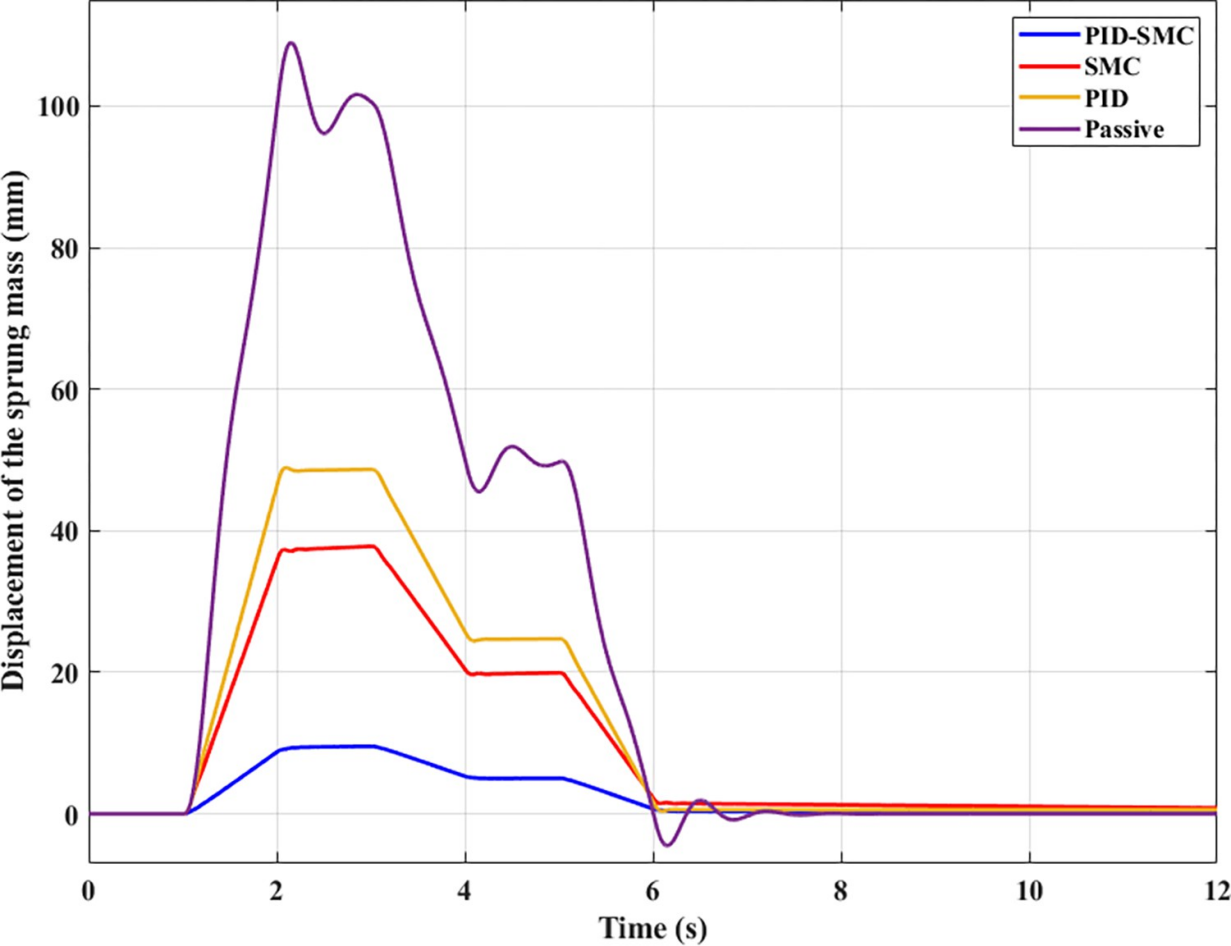
Sprung mass displacement (the second case).

The displacement of a vehicle body also obeys the changing law of the excitation signal from the road surface (Fig 9). According to this result, the magnitude of displacement decreases in order: Passive, PID, SMC, and PID-SMC. Similar to the above case, the difference between the Passive and PID-SMC scenarios is quite significant, while the difference between the other two situations is more minor. Besides, the graph of PID-SMC is smoother than that of PID or SMC. This can be understood that changing the displacement when an automobile uses an active suspension system directed by PID-SMC solution is higher comfort. At time t = 6 (s), the excitation signal from the road surface will end, and the vehicle body will oscillate continuously for a short time (Passive). For the single SMC situation, the value of the displacement is only approaching zero, while this value of the PID-SMC has completely returned to zero. Once again, the performance and stability of the hybrid algorithm are more clearly demonstrated.

**Fig 10:**
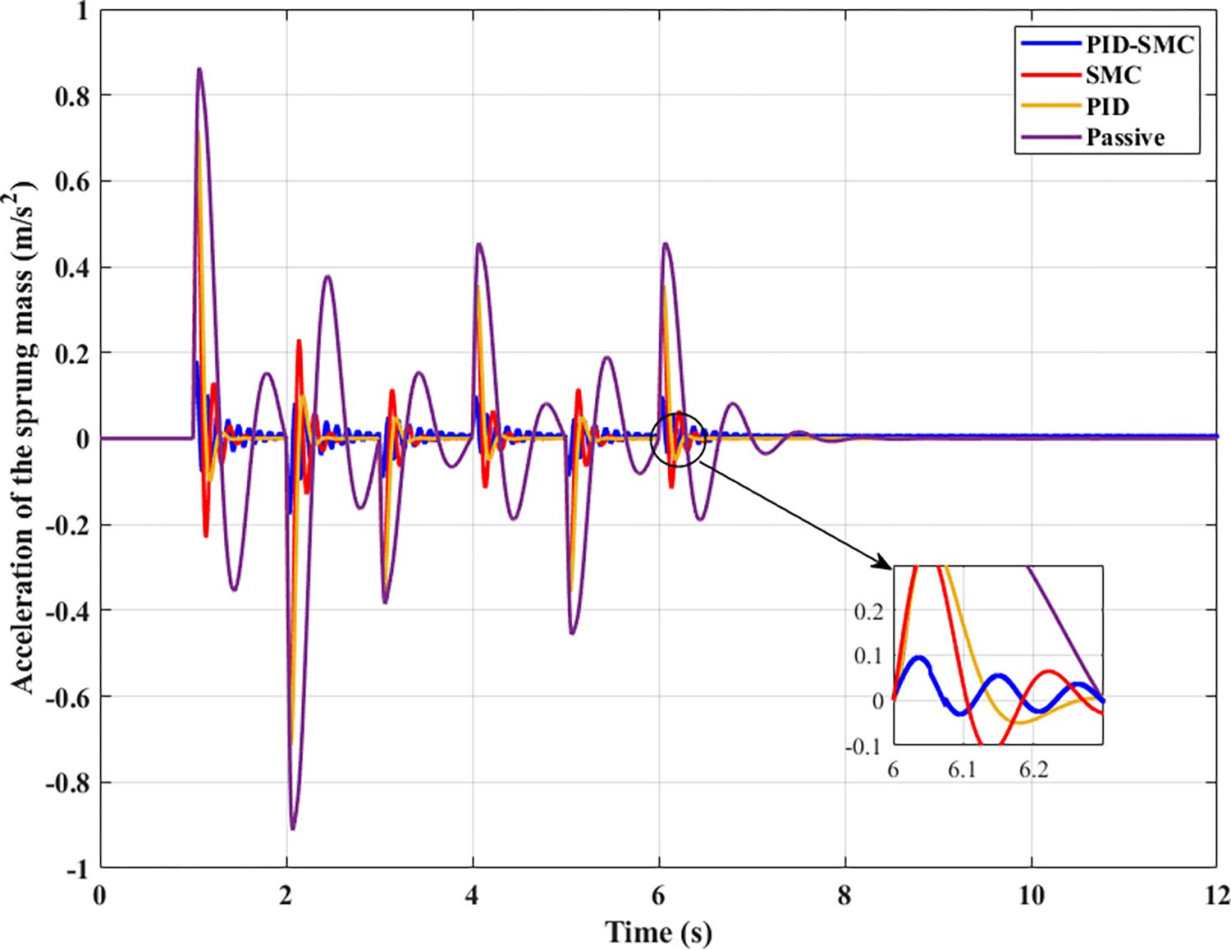
Sprung mass acceleration (the second case).

The change in sprung mass acceleration is illustrated in Fig 10. This value peaks at 0.91 (m/s^2^) if an automobile uses only a passive linear suspension system. This figure can be reduced to 0.71 (m/s^2^) once an active suspension system with the PID method is utilized to control the system. If the SMC algorithm replaces the PID algorithm, this value can be further reduced to 0.68 (m/s^2^). However, this change is quite small. Finally, if a hybrid PID-SMC solution is utilized to control the system, the maximum displacement value can be drastically reduced to only 0.18 (m/s^2^). Compared with the first situation, the displacement value in the last situation is only 19.78%, considered a relatively excellent vibration value.

From the time t = 6 (s) onwards, even though the excitation signal has ended, the vehicle body acceleration continues to change for a certain period. For PID-SMC, the change of acceleration is relatively small; this value fluctuates around the equilibrium position. Meanwhile, the acceleration values of the remaining situations fluctuate more strongly with the large amplitude.

**Fig 11:**
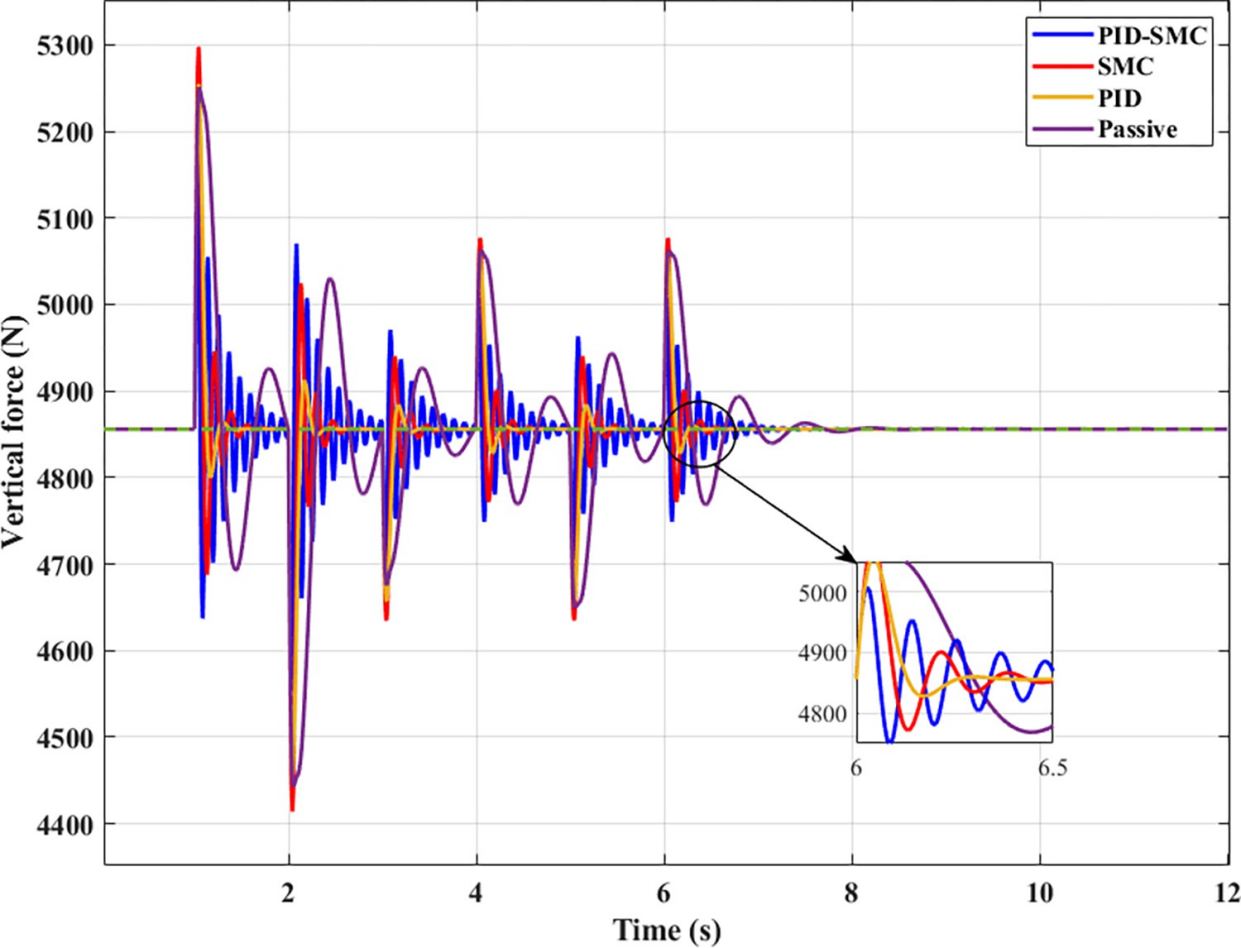
The wheel dynamic load (the second case).

The change in the dynamic load at the wheel is similar to the change in the acceleration (Fig 11). The most significant change is still in the Passive situation, and the minor change is in the SMC-PID situation. In general, if the PID-SMC hybrid algorithm is used for the suspension system, the automobile’s ride comfort and road holding are improved.

The simulation procedure outcomes are presented in Tables 2 and 3, respectively.

**Table 2:** Simulation results (the first case).

	PID-SMC	SMC	PID	Passive
Maximum displacement value (mm)	9.13	37.08	48.65	124.13
Average displacement value (mm)	5.73	23.33	30.68	71.04
RMS displacement value (mm)	6.38	25.97	34.16	79.39
Maximum acceleration value (m/s^2^)	0.54	2.05	2.13	2.57
Average acceleration value (m/s^2^)	0.06	0.22	0.29	0.68
RMS acceleration value (m/s^2^)	0.07	0.27	0.34	0.79
Minimum vertical force value (N)	4182.3	4291.6	4594.2	3843.0
Average vertical force value (N)	4855.5	4854.7	4854.3	4853.9
RMS vertical force value (N)	4856.3	4857.4	4857.9	4869.2

**Table 3:** Simulation results (the second case).

	PID-SMC	SMC	PID	Passive
Maximum displacement value (mm)	9.54	37.80	48.91	108.98
Maximum acceleration value (m/s^2^)	0.18	0.68	0.71	0.91
Minimum vertical force value (N)	4553.2	4414.1	4457.1	4442.7

In Table 2, the values mentioned include displacement, acceleration, and force values. They are considered in three states: maximum value, average value, and RMS value. Generally, the value of the PID-SMC situation is the smallest of all three states. The value of the SMC situation is larger than that of the PID-SMC, while the value of the PID situation is even larger. Finally, the values obtained for the Passive situation are the largest. This helps to demonstrate that the PID-SMC algorithm helps to ensure the ride comfort (by reducing vehicle body displacement and acceleration) and maintain road holding capacity (the change in dynamic force is negligible). Table 3 considers only the maximum values because the oscillating excitation exists only from time t = 1 (s) to time t = 5 (s). Compared to a car using only conventional mechanical suspension, if the PID-SMC algorithm is applied, its maximum displacement has been reduced by 91.25%. Besides, the maximum body acceleration is only 19.78% compared to the Passive situation. Although the change in dynamic force is more significant than in the first case, it does not affect the car’s stability much.

According to the simulation results, the performance of the PID-SMC algorithm is stable when compared with conventional single PID or SMC algorithms. Applying these algorithms has a long-lasting effect on ensuring oscillation and maintaining wheel holding. This is an essential advantage of the algorithm established in this study. However, the PID-SMC solution still has some disadvantages, such as a more complicated design process than traditional control algorithms, a more significant number of parameters that require more precise selection, the chattering phenomenon with a small amplitude still occurring, etc. In addition, some effects of disturbances and noise that cause effects in the real-time domain can degrade the quality of the system. Besides, the actual signal synthesis of the two controllers may differ slightly from the simulation. This should be verified by future specific experiments.

## 4. Conclusions

There are several causes of automobile vibration, among which the stimuli from the road surface are the leading cause of this problem. Vibrations of a vehicle can detract from the smoothness and comfort of users. Besides, if the vehicle is shaken too strongly, the interaction between the wheels and the road surface is not guaranteed. In order to improve this phenomenon, the active suspension system is equipped on the vehicle to enhance the smoothness and stability of an automobile when moving. In this article, the dynamic model has been established to simulate automobile vibration. Besides, a hybrid algorithm PID-SMC has also been designed to control the active hydraulic suspension system. The simulation process is performed by the MATLAB software with two specific cases corresponding to two types of excitation signals from the road surface. In each case, four scenarios were examined to compare the results obtained.

According to the work results, vehicle body displacement and acceleration values were significantly reduced if the PID-SMC hybrid algorithm was used to direct the active hydraulic suspension system. In addition, the change in the dynamic load at the wheel is also quite small. Therefore, this new algorithm can improve the automobile’s smoothness and road holding. In addition, the "chattering" phenomenon does not occur when combining PID and SMC algorithms, which is also considered an advantage of the new algorithm. However, this algorithm is designed to respond only to low-frequency vibrations. For high-frequency vibrations, it is necessary to improve this algorithm by incorporating some more intelligent algorithms such as Fuzzy Logic, Neural Networks, etc.

## Supporting information

S1 File (RAR)
